# The Effects of Caloric Restriction and Clinical Psychological Intervention on the Interplay of Gut Microbial Composition and Stress in Women

**DOI:** 10.3390/nu16162584

**Published:** 2024-08-06

**Authors:** Luise Bellach, Alexandra Kautzky-Willer, Kathrin Heneis, Michael Leutner, Alexander Kautzky

**Affiliations:** 1 Department of Internal Medicine III, Division of Endocrinology and Metabolism, Medical University of Vienna, 1090 Vienna, Austria; 2 Department of Psychiatry and Psychotherapy, Medical University of Vienna, 1090 Vienna, Austria

**Keywords:** gut microbiome, caloric restriction, mental health, psychological stress, very-low-calorie diet, F.X. Mayr diet, clinical psychological intervention, multilevel dimension reduction, women’s health

## Abstract

Both mental and metabolic disorders are steadily becoming more prevalent, increasing interest in non-pharmacological lifestyle interventions targeting both types of disorders. However, the combined effect of diet and psychological interventions on the gut microbiome and mental health outcomes remains underexplored. Thus, in this study, we randomized 41 women into two caloric restriction (CR) dietary groups, namely very-low-calorie diet (VLCD) and F.X. Mayr diet (FXM). The patients were then further randomized to either receive clinical psychological intervention (CPI) or no CPI. Blood and fecal samples were collected before and after two weeks of CR. Psychometric outcomes were assessed using the Perceived Stress Scale (PSS), Brief Symptom Index (BSI), and Burnout Dimension Inventory (BODI). Stool samples underwent 16S-rRNA sequencing. Upon two weeks of CR, α-diversity decreased overall and longitudinal PERMANOVA models revealed significant shifts in β-diversity according to diet, CPI, age, and body-mass-index. Furthermore, *Agathobacter*, *Fusicatenibacter*, and *Subdoligranulum* decreased in abundance. However, the *Oscillibacter* genus was enriched solely in FXM. CPI had a negligible effect on the microbiome. Dimension reduction models revealed clusters of taxa which distinctly associated with psychometric outcomes. Members of the *Oscillospiraceae* family were linked to favorable psychometric outcomes after two weeks of CR. Despite α-diversity reductions after CR, enrichment of *Oscillospiraceae* spp., solely seen in FXM, correlated with improved psychometric outcomes. This study suggests a promising direction for future interventions targeting mental health through gut microbial modulation.

## 1. Introduction

Common mental disorders such as depression, as well as metabolic disorders such as obesity and diabetes, rank among both the most prevalent and most burdensome diseases. Concerningly, these disorders were shown to share common biological as well as lifestyle pathways [1], leading to frequent co-occurrence [2] and even higher morbidity and mortality [3]. While effective pharmacological treatment is available for these metabolic and mental disorders, sufficient adherence is challenging for patients with both diabetes and depression. Furthermore, side effects such as weight gain are common and can further strengthen the link between obesity, diabetes, and mental disorders [4].

This has led to increased interest in lifestyle interventions, which can be effective and well-tolerated among both patients with obesity and diabetes [5,6] as well as mental disorders [7]. A stressful lifestyle and poor diet can harm both mental and physical health [8]; however, only in recent years did scientific advancement point towards the gut microbiome as a possible mediator. The term gut microbiome entails the multitude of bacteria, viruses, fungi, and other microbes like protozoans and archaea resident in the gastrointestinal tract [9], which interact with the host in a bidirectional manner [10]. More specifically, dietary choices like the ingestion of saturated fatty acids or amount of fiber influence the composition of the gut microbiome [10]; however, intermediary metabolites of the gut microbes have also been shown to influence the somatic and mental health of the host [9,10]. For example, it has been shown that the gut microbiome modulates anxious and social behavior by influencing corticosterone levels and neurotransmission in specific brain regions like the amygdala or the hippocampus [11,12]. Thereby, as diet inadvertently affects the gut microbiome, the relationship between gut microbiota and stress has bidirectional characteristics as stressful events can directly alter gut microbial composition [13]. This so-called microbiome–gut–brain axis consists of a systemic exchange between gut microbes and the central nervous system via gut-peptides, microbe-associated circulating molecules, immune regulation, tryptophane metabolism, and the vagus nerve [14,15]. 

Along these lines, specific microbial profiles and related measures such as diversity indices have recently been linked to mental disorders such as depression and anxiety disorders [16], and may bear potential for therapeutic interventions. Various dietary protocols have been shown to modulate gut microbiota and improve physical health and cognitive function [17,18,19,20]. In particular, caloric restriction (CR), i.e., drastically reducing the daily calorie intake, has immediate effects on gut microbial composition as well as mental wellbeing [21]. We have previously reported two CR regimens, a very-low-calory diet (VLCD) or a VLCD with oral magnesium supplementation in accordance with the F.X. Mayr diet protocol (FXM), to be effective in reducing obesity and enhancing mental wellbeing in women with obesity and psychological stress [22]. However, the data on CR and gut microbiome is yet heterogenous with one study pointing towards an increase in α-diversity [23] while another noted a reduction of bacterial abundance and an increase in Clostridium difficile [24], similar to findings in inflammatory bowel disease [25]. Conflicting findings may be owed to differential effects of various CR protocols on microbiome and mental wellbeing that are still poorly understood. For example, micronutrients such as magnesium influence gut microbial communities. Mouse experiments showed that magnesium deficiency disrupts gut microbial composition and is associated with anxiety and depression-like behavior [26,27]. Conversely, oral supplementation of magnesium sulfate was linked to inflammation-specific changes in gut microbiota [28]. A comprehensive assessment on the effect of magnesium supplementation, which is part of some CR protocols, has not been published in humans thus far. 

However, not only diet but also stress affects the gut microbiome. Alleviation of stress not only augments physical and mental health in obesity [29] but also leads to alterations in gut microbial composition. Being a standard treatment for common mental health disorders as well as proven effective in supporting outcomes in patients with metabolic disorders such as obesity and diabetes, psychotherapeutic interventions may therefore be a promising addition to dietary measures. For instance, in patients with inflammatory bowel disease, a combined protocol with diet and psychotherapeutic interventions was associated with changes in gut microbiome related positively with mental wellbeing [30]. However, the question of whether the effects elicited from dietary intervention and stress modulation act independently or synergistically and the role of specific dietary protocols remains yet to be tackled. 

Thus, our prospective clinical trial involves women randomized into two dietary groups, featuring different modes of CR with and without magnesium supplementation, alongside psychological interventions. This study aims to identify distinct shifts in microbial composition and understand how the gut microbiome relates to psychometric parameters. 

## 2. Materials and Methods

The study population consists of 41 women, detailed in Table 1, who completed two weeks of inpatient lifestyle intervention at the “La Pura Women’s Health Resort”. Thereby, a dietary intervention of CR was combined with a clinical psychological intervention (CPI). Exclusion criteria were current pregnancy or severe chronic illness. The participants were free to use La Pura’s leisure and wellness facilities independently of their respective randomization group. All procedures were approved by the ethics committee of Lower Austria. A more detailed description of the study population regarding clinical characteristics and health outcomes has been published previously [22]. The study was registered at ClinicalTrials.gov under the accession number NCT04848948 and the flowchart of the study can be seen in Figure S6.

### 2.1. Intervention Groups

All patients were randomized to receive VLCD or FXM. Both diets were based on individualized meal plans according to macronutrient percentage and calorie limits. VLCD consists of a caloric restriction of 630–700 kcal/d, split up into three meals, each consisting of 20% fat, 34% protein, and 46% carbohydrates [22,31]. Compared to this, FXM consists of combining caloric restriction (i.e., 700–800 kcal/d, split into two meals consisting of 26% fat, 21% protein, and 53% carbohydrates) with the additional rules of prolonged food chewing, no meals after seven p.m., and mixed meals with 2 × 120 mg oral magnesium sulphate supplementation to increase gastrointestinal peristaltic propulsion and bile acid production while dampening insulin peaks. Additionally, abdominal massages are used to boost peristalsis [22,31]. No further micronutrients were supplemented or analyzed. Furthermore, within each dietary group, all patients were again randomized to either receive CPI or a single lecture on stress prevention held by a clinical psychologist. The CPI consisted of two sessions of muscle relaxation and mindfulness training (Jacobson), two one-on-one psycho-educative sessions focusing on stress prevention, and three sessions of biofeedback, totaling seven sessions of 50 min [22,31].

### 2.2. Clinical Variables and Questionnaires

Clinical variables included age, body-mass-index (BMI), C-reactive protein (CRP), morning cortisol from sputum, and interleukin 6 (IL-6). Psychometric measurements were taken at baseline and at the follow-up check-in with the study team after 14 days. The questionnaires employed were the Perceived Stress Scale (PSS) [32], the Brief Symptom Index (BSI) [33], and the burnout dimension inventor (BODI) [34]. As previously described, the participants decreased in both BMI and PSS [22]. Specifically, mean BMI change was −0.80 ± 0.52 and mean PSS change was −3.38 ± 4.69, with 44.74% of participants reaching a decrease by a minimum of 3 points.

### 2.3. Biological Specimen Sampling

Fasting blood samples to assess CRP and IL-6 levels were drawn at baseline and at follow-up. Sampled stool (at baseline and at follow-up) was stored at −20 °C until all study samples were collected.

### 2.4. 16S rRNA Sequencing and Microbial Data Processing

All stool samples were processed at the JMF Vienna according to their standardized pipeline [35] and sequencing was performed via Illumina. Reads were filtered and trimmed. Denoising was performed with DADA2.

### 2.5. Statistical Analysis

All statistical analyses were performed in R version 4.3.1. With the exception of descriptive presentation of microbial composition and α-diversity indices, only taxa present in at least 10% of samples were included into the analyses. To reduce the risk of including spurious taxa into the analyses, any taxon with a count of 2 or less per sample was excluded from analysis. Microbial data were stored in a phyloseq object and handling of taxonomic data was performed through the *microViz* package. The choice of an appropriate data processing pipeline was aided by Gloor et al. [36]. Prior to data transformation via additive-log ratio, all zero counts were offset with the value 1. Figure panels were compiled through the *ggpubr* package and *Inkscape*. Due to the explorative nature of the study, a hypothesis-based *p*-value correction approach was chosen. The full code used for this analysis can be found on GitHub (https://github.com/luisebe/diet-mental-health-microbiome, accessed on 2 August 2024).

#### 2.5.1. Description of Microbial Composition

Microbial composition, expressed as relative abundance on genus level, was plotted via the *microViz* pipeline, respectively, for each diet group. Ranks of the 10 most common genera, respectively, at baseline and follow-up were displayed as Sankey plots using *ggalluvial* (Version 0.12.5). Further, a multilevel principal component analysis (PCA) using two components defined visually via screeplot was performed via the *mixOmics* (Version 6.26.0) package after correction for within-sample variation.

#### 2.5.2. Diversity Indices

α-diversity was assessed via the *microViz* (Version 0.10.10) package by microbial richness, Shannon’s index, and the Firmicutes/Bacteroidetes ratio. To assess baseline differences, for each index an unpaired *t*-test was applied with diet as the grouping variable. Changes between baseline and follow-up were assessed via repeated measures ANOVA to account for BMI categories (normal-weight, overweight, and obesity with BMI < 25 kg/m^2^, BMI 25–30 kg/m^2^, and BMI >= 30 kg/m^2^, respectively). β-diversity was computed for each timepoint via Aitchison distance, as featured in the *microViz* (Version 0.10.10) package. PERMANOVA models assessing beta-diversity longitudinally were computed via the *vegan* (Version 2.6-4) package. Thereby, sequential models were built starting from a main effect of timepoint and adding, respectively, diet, CPI, age, and BMI as well as their interactions. To further elucidate significant effects of diet, the absolute difference in β-diversity between the two time-points was assessed via the unpaired *t*-test and displayed graphically via boxplots. Finally, β-diversity was plotted cross-sectionally after CR with principal coordinates analysis (PCoA) using the *microViz* (Version 0.10.10) pipeline. When testing for significance, *p*-value adjustment was performed for each set of tests according to the Benjamini–Hochberg method. A significance cut-off value of 0.05 was chosen.

#### 2.5.3. Longitudinal Changes of Taxa

Longitudinal differential abundance analyses were performed via the *ANCOMBC* (Version 2.4.0) package. Respectively, on family, genus, and species level, for each taxon a mixed-effects model was employed assessing the fixed effect of timepoint while using the participant identifier as a random effect. The analysis on genus level was then repeated after stratification by diet and, subsequently, by diet and CPI intervention group. The log-fold changes were plotted via bar and tile plots, using *ggplot2* (Version 3.4.4), connected dot plots, using *microbiomeutilities* (Version 1.00.17), and violin plots, using *MicrobiomeStat* (Version 1.3.3). When testing for significance, *p*-value adjustment was performed according to the ANCOM BC2 pipeline, adjusting for the number of effects, respectively, on family, genus, and species level. A significance cut-off value of 0.05 was chosen.

#### 2.5.4. Multilevel Dimension-Reduction Analyses

A regressional multilevel sparse partial least squares analysis (sPLS2) with an outcome dataset was performed, integrating both microbial data on the genus level and clinical data to assess how gut microbial composition influences psychometric variables as well as somatic parameters detailed in Table 1. The *mixOmics* (Version 6.26.0) package was used after correction for within-sample variance, reduction of near-zero variance, and imputation of missing clinical data using the NIPALS algorithm. The optimal model was chosen according to feature selection performed via 5-fold cross-validation repeated 100 times. The variables were plotted in a correlation circle plot together with the weights of the loading vectors on component 1 to obtain a clear picture on the data set. Finally, the variables explaining the first component of the sPLS2 output were plotted in a clustered image map to present the correlations between the microbial and clinical variables in an intuitive manner. Next, sPLS discriminatory analysis (sPLS-DA) was performed for binary classification of subjects receiving FXM and VLCD, as well as those with successful and unsuccessful stress reduction (PSS score change ≥ 3). Optimal models were selected by feature selection performed via 3-fold cross-validation repeated over 1000 times. Performance assessment was conducted via the classification error rate according to maximal distance, as suggested in the *mixOmics* workflow [37].

## 3. Results

### 3.1. Microbial Composition

**Figure 1:**
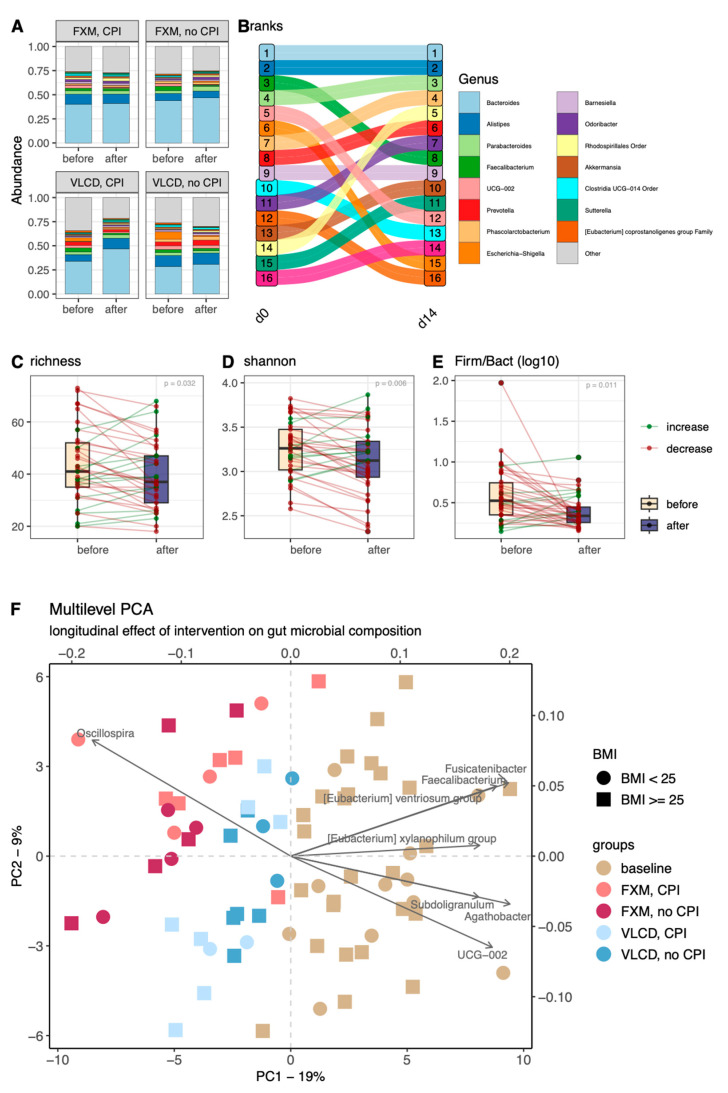
Overview of taxonomical baseline characteristics and changes over the intervention period. (**A**) Relative abundances across the intervention groups; (**B**) relative changes in abundance ranks in the overall cohort; (**C**–**E**) changes in bacterial richness, Shannon’s index, and Firmicutes/Bacteroidetes ratio; (**F**) multilevel PCA biplot colored according to baseline (beige), FXM (light pink = with CPI, dark pink = without CPI), and VLCD (light blue = with CPI, dark blue = without CPI) and taxonomy (grey arrows) as well as BMI category (shapes). FXM—F.X. Mayr diet, VLCD—very-low-calorie diet, CPI—clinical psychological intervention, BMI—body-mass-index.

An overview of gut microbial composition per participant, stratified by timepoint and diet group, is presented in Figure 1A. Looking at compositions of relative abundance at genus level, Bacteroides and Alistipes were, respectively, the first and second ranked genera throughout the study period (Figure 1B). *Faecalibacterium*, *UCG-002*, *Escherichia-Shigella*, and *Clostridia UCG-014* and *E. coprostanoligenes* decreased in relative abundance ranks, while *Rhodospirillales*-Order, *Parabacteroides*, *Phascolarctobacterium*, *Prevotella*, *Odoribacter*, and *Akkermansia*, increased. Changes in α-diversity indices are shown in Figure 1C–E. The overall longitudinal changes in microbial composition are depicted in a multilevel PCA in Figure 1F. The cluster containing fecal samples at baseline is visibly separated from the clusters of follow-up samples, corresponding to a negative shift alongside the first principal component of the PCA (PC1) with FXM samples clustering farther along the negative scale. The most important determinant taxa for sample clustering along PC1 are *Oscillospira*, which is negatively associated with PC1, and *Agathobacter*, *Subdoligranulum*, *Fusicatenibacter*, *E. xylanophilum*, *E. ventriosum*, *Roseburia*, *UCG-002*, and *Faecalibacterium*, which are positively associated with PC1 (Figure 1F).

### 3.2. Diversity Indices

Regarding α-diversity, groups differed in richness, Shannon’s index, and Firmicutes/Bacteroidetes ratio prior to CR. All indices decreased significantly over time (all *p* < 0.05, Figure 1C–E). Regarding β-diversity, the best performing PERMANOVA model included significant effects of timepoint in interaction with diet, as well as CPI and age (R^2^ = 0.12, *p* < 0.05; Table 2). More pronounced absolute changes in β-diversity were observed in FXM compared to VLCD (*p* = 0.01; Figure S1). After the intervention, samples were separated along the first component by diet, as visualized via a PCoA (Figure 4C).

### 3.3. Longitudinal Changes in Relative Abundance

**Figure 2:**
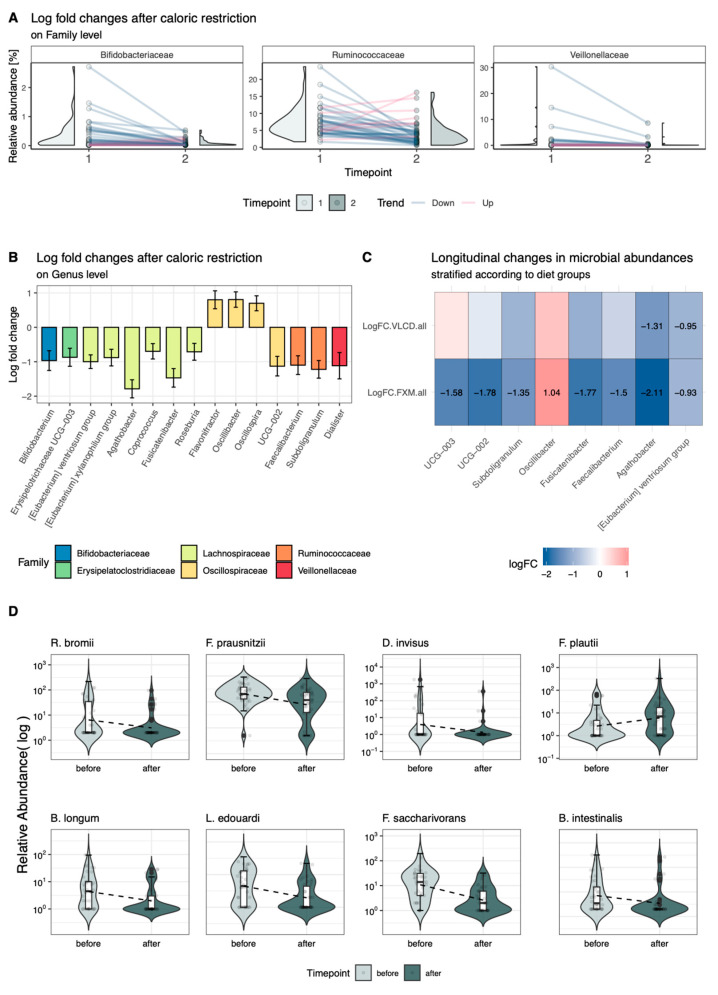
Mixed linear effects models of longitudinal microbial changes across the intervention period. (**A**,**B**) Significant log-fold changes in relative abundances on family (**A**) and genus (**B**) level; (**C**) log-fold changes in relative abundance on genus level and stratified according to diet and dietary subgroups–significant changes are annotated with their respective log-fold change; (**D**) significant log-fold changes in relative abundance on species level in the overall cohort–depicted according to diet (pink = FXM, blue = VLCD). FXM = F.X. Mayr diet, VLCD = very-low-calorie diet; *p*-value adjustment was performed via Benjamini–Hochberg’s method. *R. bromii* = *Ruminococcus bromii*, *F. prausnitzii* = *Faecalibacterium prausnitzii*, *D. invisus* = *Dialister invisus*, *B. longum* = *Bifidobacterium longum*, *L. edouardi* = *Lachnoclostridium edouardi*, *F. saccharivorans* = *Fusicatenibacter saccharivorans*, *B. intestinalis* = *Bacteroides intestinalis*, *F. plautii* = *Flavonifractor plautii*.

Six families decreased significantly in relative abundance following CR, including *Bifidobacteriaceae* and *Ruminococcaceae* (adj. *p* < 0.05, Figure 2A). Significant decreases on genus level included *Bifidobacterium*, *Dialister*, *Faecalibacterium*, and *Subdoligranulum* (Figure 2B). Genera of the *Lachnospiraceae* family decreased in abundance (*E. ventriosum* group, *E. xylanophilum* group, *Agathobacter*, *Coprococcus*, *Fusicatenibacter*, and *Roseburia*). Among the *Oscillospiraceae* family, both increasing (*Flavonifractor*, *Oscillibacter*, and *Oscillospira*) and decreasing (*UCG-002*) genera were observed (Figure 2B). Stratifying the data according to intervention groups revealed patterns primarily according to diet (Figure 2C). While *Agathobacter* and *E. ventriosum* group consistently decreased across both groups, decreases in *UCG-003*, *UCG-002*, *Subdoligranulum*, *Fusicatenibacter*, and *Faecalibacterium* reached significance only in FXM. Interestingly, solely in FXM there was a significant increase in the generus of *Oscillibacter* to be seen. Further stratification according to CPI subgroups yielded no significant results upon *p*-value adjustment. When aggregating the taxa on species level, significant longitudinal decreases in *R. bromii*, *F. prausnitzii*, *D. invisus*, *B. longum*, *L. edouardi*, *F. saccharivorans*, and *B. intestinalis* as well as increases in F. plautii were seen in the overall collective, but not in the diet subgroups (Figure 2D). Further stratification according to dietary and CPI subgroups reflected the overall trend seen in Figure 2C yet did not remain significant after *p*-value adjustment (Figure S5).

### 3.4. Multilevel Dimension-Reduction Analyses

**Figure 3:**
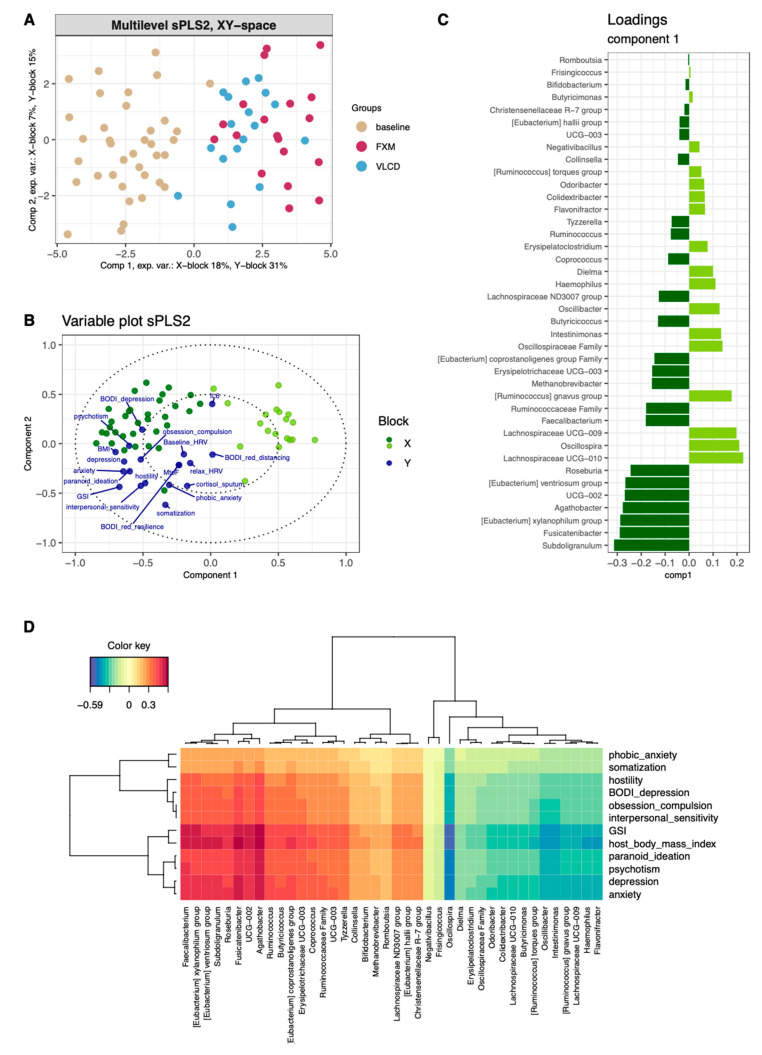
Longitudinal multilevel sPLS2 model of taxonomical and clinical parameters. (**A**) sPLS2 model colored according to baseline (beige) and diet (pink = FXM, blue = VLCD); (**B**) variable plot of the sPLS2 model depicting the clustering of both the microbial data (Block X = green) and the clinical data (Block Y = dark blue)–microbial data were further colored according to the loadings for component 1 (light green = positive, dark green = negative); (**C**) loadings plot of microbial data along component 1 of the sPLS2 model (light green = positive, dark green = negative); (**D**) clustered image map depicting the associations between both taxonomical and clinical variables along component 1 of the sPLS2 model. FXM = F.X. Mayr diet, VLCD = very-low-calorie diet.

**Figure 4:**
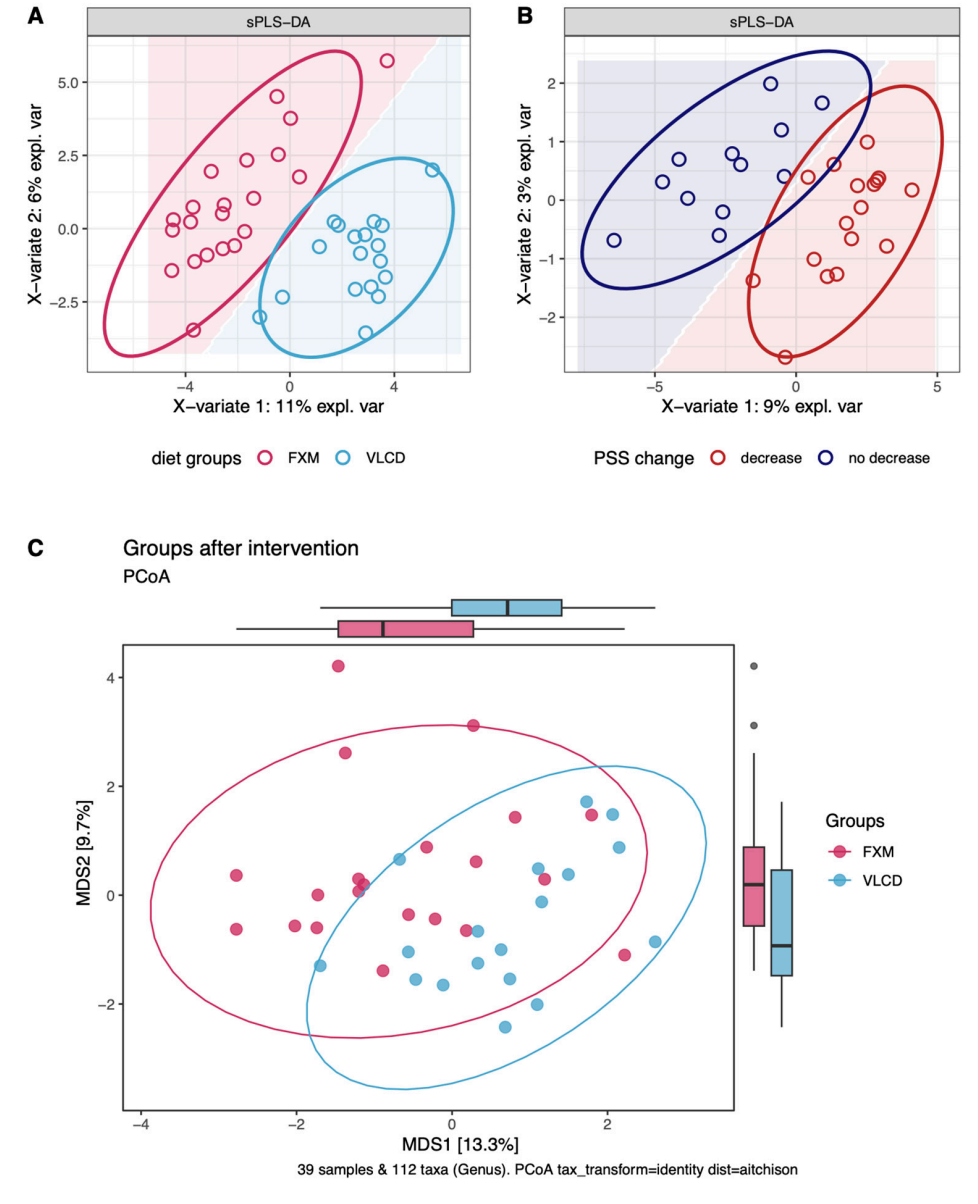
Exploration of taxonomic composition after the intervention period through dimension reduction. (**A**) sPLS-DA model for the classification of diet type (pink = FXM, blue = VLCD); (**B**) sPLS-DA model for the classification of successful PSS reduction (brown = decrease in PSS, dark blue = no decrease in PSS); (**C**) PCoA colored according to diet (pink = FXM, blue = VLCD). FXM = F.X. Mayr diet, VLCD = very-low-calorie diet, PSS = perceived stress scale.

The sPLS2 selected 40 taxa (“X-space”) and 12 clinical variables (“Y-space”) to optimally explain the data set. Samples could primarily be distinguished along the first component of the XY space according to sampling timepoint, with FXM more positively associated with the first component than VLCD (Figure 3A). The most informative parameters determining the position along the first component are displayed in Figure 3C (taxa) and Figure 3B (clinical parameters). Clustering microbial and clinical variables contributing to the first component revealed two sets of microbial genera. The first cluster was characterized by positive association with psychometric scores and BMI, while the second cluster showed the reversed pattern (Figure 3D). Strongest positive correlations with clinical parameters in cluster 1 involved *Agathobacter*, *Fusicatenibacter*, and *Subdoligranulum*, while the strongest negative correlation with clinical parameters in cluster 2 was observed in *Oscillospira*. The exploratory sPLS-DA model for classification of diet selected 70 taxa as informative. FXM and VLCD were separable with an error rate of 0.2 obtained by cross-validation (Figure 4A). The five most informative taxa driving this classification along the first component were *Subdoligranulum*, *UCG-003*, *R. torques* group, *Lachnospiraceae*, and *Erysipelatoclostridium* (Figure S4A). The exploratory sPLS-DA for classification of successful stress reduction revealed a moderate separation alongside the first component (Figure 4B). The corresponding loading plot revealed that the separation of the model was mainly driven by *Bifidobacterium*, *E. siraeum*, *Hydrogenoanaerobacterium*, *Lachnospiraceae-UCG-001*, and *Oscillospirales* (Figure S4B).

## 4. Discussion

Following two weeks of caloric restriction, gut-microbiome composition in a mixed sample of women who were overweight and experiencing psychological stress was altered in dependence of dietary protocols and psychological interventions. In particular, the FXM diet caused distinct microbial shifts headlined by an increased abundance of *Oscillibacter* that were linked to good mental health and a successful reduction of psychological stress. Further stratification into the CPI subgroups did not yield additional effects on the microbial composition.

We have previously shown in this sample that short-term CR is favorably linked to metabolic and mental health [22]. Microbial composition may thereby mediate anti-inflammatory properties, regulation of hypothalamus pituitary–adrenal axis trafficking, and eventually preservation of hippocampal neurons associated with CR [38]. An immediate yet reversible effect of CR on intestinal microbiota was reported previously [21,39,40]. While scientific data on FXM diet remain scarce, patterns of microbial shifts in VLCD have been analyzed in mouse [39,40,41] and human cohorts [24,42,43,44,45], albeit to inconsistent results. We observed mostly decreases in relative abundance upon CR regimens, predominantly in the phylum *Firmicutes* and the *Lachnospiriceae/Ruminococcaceae* family. This is in line with previous reports of a reduced Firmicutes/Bacteroidetes ratio following CR regimens [46]; however, the relevance of this metric has been questioned by recent insights in favor of fine-grained analysis on genus and species level. 

On the genus level, *Lachnospiriceae* family members *Agathobacter* and *Eubacterium ventriosum* group declined, in line with previous findings in subjects with obesity undergoing VLCD and intermittent fasting [24,47,48]–these findings held true when stratifying according to diet group. Furthermore, *Roseburia*, *Coprococcus,* as well as *Ruminococceae* family members *Faecalibacterium* and *Subdoligranulum,* also depleted during intervention, agreeing with clinical trials applying, respectively, 4 days of low-calorie and protein-rich diet and 8 weeks of VLCD [21,24]. Depletion of *Coprococcus*, *Faecalibacterium,* and *Subdoligranulum* is mimicking potential endotypes of depression [49], which may serve as transdiagnostic markers of psychopathology [16]. *Faecalibacterium* [50] and *Roseburia* [51], in particular, are main sources of short chain fatty acids (SCFAs) butyrate and propionate which were linked to inflammation and neurotransmitter signaling [52]. Of note, when stratifying according to dietary group, only the FXM group showed a significant depletion of *Faecalibacterium* and *Subdoligranulum.* Interestingly, also exclusive for the FXM group, there was an increase in *Oscillibacter*, a genus which has recently been linked to an improved cardiometabolic risk profile by lowering cholesterol [53]. On the species level, a possible depletion of butyrate-producing taxa can also be seen in the decreased abundance of *R. bromii* which has been associated with increased fecal butyrate levels [54]. 

However, microbial shifts during CR may have unfavorable health implications. On the other hand, common symptoms of affective disorders include reduced appetite and weight loss which may produce shifts in microbiome composition, partly mimicking CR. Along these lines, *Roseburia*, *Faecalibacterium,* and *Subdoligranulum* were found depleted while other *Lachnospiraceae* family members were enriched in anorexia nervosa patients [55,56]. Irrespective of these considerations, our results do not support short-term causal links between these SCFA producing microbiota and mental wellbeing considering that both microbiota and psychometric symptom scores declined during CR. 

In contrast, *Akkermansia* of phylum *Verrucomicrobiota* and *Odoribacter* of phylum *Bacteroidetes* showed positive selection during CR and passively entered the top ten most abundant genera as other taxa were diminished. Both genera were consistently linked to positive mental- and metabolic-health outcomes [57,58]. In particular, *Akkermansia* was previously shown to increase during CR [24,42,44] and potentially even long term [43]. Interestingly, *Akkermansia* was attributed a gatekeeping role of microbial colonization due to the ability to metabolize host glycans and regulate gut mucosa integrity, which fits well with the patterns observed here. *Odoribacter* was attributed a regulatory role of succinate, an intermediary propionate metabolite, and a pro-inflammatory endotype with low *Odoribacter* contrasted by high succinate-producing *Prevotellacaea* and *Veillonellaceae* families was associated with obesity and type-2 diabetes mellitus [59]. In accordance with this theoretical framework, here the overall successful reduction of BMI as well as stress correlates was accompanied by decreasing abundance of genera *Dialister* of *Veillonellaceae* relative to *Odoribacter* and *Akkermansia*. 

Further increases in microbial abundance upon CR were observed in species of the *Oscillospiraceae* family, such as *Flavonifractor*, *Oscillospira,* and *Oscillibacter*–the latter was significant exclusively in FMX. This microbial shift relates to a cluster of genera identified by multilevel sPLS2 that was negatively associated with psychometric scores. *Oscillibacter* has been shown to be enhanced in depression [60,61]–interestingly, *Oscillibacter* produces valeric acid as metabolic byproduct, a molecule which can bind to the GABA-A receptor [61,62]. As recent research has pointed toward GABA signaling as a possible additional pharmacologic target in depressive disorders [63], it remains to be determined whether this positive association of *Oscillibacter* with depression might in fact be counter-regulatory. Nevertheless, contrary to associations of *Oscillibacter* and *Flavonifractor* with depression [60,61,62,64,65], including *F. plautii* [66], *Oscillospira* was recently suggested as a target for novel probiotic treatment due to association with beneficial metabolic phenotypes such as leanness and low inflammation [67]. Along these lines, and fitting well with a mouse stress-exposure model, successful stress reduction was predicted by higher abundance of *Akkermansia* and *Oscillospira* as well as lower abundance of *Roseburia* [68]. Thus, our findings are indicative of some taxa relating to stress unidirectionally and other taxa, such as *Oscillospira*, representing promising bidirectional targets regarding psychological stress that can be separated as clusters by multilevel sPLS2 and sPLS-DA. In fact, *Akkermansia* has already been shown to improve stress-induced depressive-like symptoms in mice [69], possibly through increased serotonin levels in the gut and the prefrontal cortex [70]. 

Regarding limitations, short-term CR interventions were applied without a no-diet control group and without accounting for long-term effects beyond two weeks. We expect representative changes despite only two weeks of CR considering that the gut microbiome is known to adapt to dietary disturbances within days [21,71]. However, long-term implications remain to be addressed, particularly in the high of microbial shifts reversing to baseline irrespective of CR duration in several trials [43,45]. More concerning, baseline intraindividual differences were shown to outnumber and withstand shifts observed during CR [45,47]. The study sample size is limited yet competitive with the literature on microbiome and CR, especially regarding longitudinal intervention trials. Although there is no sex-specific difference which needs to be accounted for, our overall sample varied in psychometric parameters, BMI, age and, consequently, in menopausal status. Furthermore, we could not adjust for diet prior to study inclusion and individual differences in usage of leisure activities at the women’s health resort. Additionally, the study was conducted in a health resort with special living circumstances, bringing along potential selection bias and limiting generalizability of our results. Despite correction for multiple testing to counter false positive findings, substantially larger datasets accounting for pre-intervention endotypes may be needed for reproducible and meaningful results. Consequently, the stratified results in FXM and VLCD in particular must be interpreted cautiously. On a speculative note, the divergent effects on microbial composition observed in the diet groups may be related to FXM, including magnesium intake. On the one hand, gut dysbiosis and adverse mental health outcomes were associated with low magnesium [26,27]. On the other hand, excess magnesium supplementation can decrease α-diversity and impair the functional integrity of the gut microbiome, leading to decreased carbohydrate energy harvesting and an increase in lipopolysaccharide production [72]. Finally, diet was based on macronutrients and calorie limits determined by individualized meal plans, disregarding effects of other micronutrients such as minerals and vitamins that may also be relevant to changes in gut microbiome.

## 5. Conclusions

To the best of our knowledge, we are the first to prospectively assess the combined effects of dietary and psychological intervention on the gut microbiome in relation to psychometric outcomes. Furthermore, our patient collective consisting of overweight and stressed women can be considered at risk of both mental and metabolic comorbidities, thus making it an appropriate target population for lifestyle interventions aimed at early metabolic and psychological benefits and disease prevention. In summary, we were able to show that a short-term caloric restriction led to perturbations in gut microbial compositions that clustered distinctly with psychometric outcomes. The taxonomic changes observed over the study period were most pronounced in the participants receiving the FXM diet while add-on clinical psychological intervention showed little additional effects. The lack of effect upon CPI might be due to the decreased sample size in the respective subgroups or due to the fact that diet-related interventions have a more direct and impactful effect on the gut microbial composition. Nevertheless, on the one hand, our results suggest *Oscillospira* as a microbial strain with psychoactive potential on psychological stress and further support a role of *Akkermansia* and *Odoribacter* in metabolic and mental health. On the other hand, depletion of some butyrate-producing bacteria such as *Faecalibacterium*, *Roseburia*, and *Subdoligranulum* advises caution. However, implications remain vague as effects of CR were shown to be dependent on calorie restriction levels and to be generally reversible even after prolonged CR regimens [41,43]. Along these lines, well-replicated associations of taxa with various health outcomes are still lacking evidence of causality. For example, despite consistent associations with various metabolic outcomes, no detectable changes in metabolic parameters were observed upon administering a cultivated strain of *Subdoligranulum* to mice [73]. Consequently, direct examination of target microbes via prospective and interventional trials are needed to avoid fallacies.

Yet despite these caveats, we provide a starting point for future interventional studies aiming to assess the efficacy of potential target microbes such as species from the genera *Oscillospira* for improving mental health outcomes like psychological stress. Together with our previous publications [22,31], we were able to show that the microbial shifts upon short-term CR were associated with beneficial changes regarding psychometric properties and body weight. Thus, we provide further support that short-term dietary interventions can strengthen both mental and metabolic health. Our results suggest that add-on psychological intervention hardly modulates the gut–brain axis; however, longer time periods may be required to elucidate additional effects to diet that should be explored by future studies. 

## Figures and Tables

**Table 1:** Baseline characteristics.

Variable	VLCD	FXM	*p*-Value
Age [years], mean ± SD	53.5 ± 10.82	53.55 ± 13.48	0.99 ^a^
Peri/postmenopause, n (%)	14 (78%)	14 (70%)	0.11 ^d^
BMI [kg/m^2^], mean ± SD	28.69 ± 5.93	27.43 ± 5.93	0.47 ^b^
CRP [mg/dL], mean ± SD	0.51 ± 0.49	0.28 ± 0.36	0.06 ^b^
Cortisol in sputum [µg/dL], mean ± SD	0.46 ± 0.34	0.54 ± 0.32	0.23 ^b^
IL6 [pg/mL], median ± IQR	1.95 ± 3.32	1.87 ± 1.95	0.80 ^c^
GSI, mean ± SD	54.06 ± 9.84	57.5 ± 10.72	0.32 ^a^
PSS, mean ± SD	13.33 ± 3.73	16.94 ± 5.07	0.04 ^a^
MtoF, mean ± SD	1.11 ± 0.24	1.09 ± 0.24	0.86 ^b^
BODI: dysfunctional compensation, median ± IQR	62 ± 96.5	45 ± 78	0.48 ^c^
BODI: reduced resilience, mean ± SD	25.18 ± 15.3	35.95 ± 19.41	0.09 ^a^
BODI: depression, median ± IQR	33.2 ± 22.9	26.9 ± 27.7	0.30 ^c^
BODI: reduced distancing, median ± IQR	69.7 ± 35.73	53.8 ± 56.4	0.66 ^c^
Baseline HRV [ms], mean ± SD	12.21 ± 13.71	15.52 ± 13.59	0.36 ^b^
Relaxed HRV [ms], mean ± SD	16.07 ± 14.59	16.3 ± 9.74	0.62 ^b^
Stressed HRV [ms], mean ± SD	16.35 ± 16.12	17.03 ± 11	0.71 ^b^
BSI: obsession-compulsion, mean ± SD	52.76 ± 9.09	55.85 ± 11.76	0.38 ^a^
BSI: phobic anxiety, median ± IQR	45 ± 10	55 ± 16	0.03 ^c^
BSI: interpersonal sensitivity, mean ± SD	53.47 ± 9.64	55.35 ± 9.54	0.56 ^a^
BSI: psychotism, median ± IQR	54 ± 10	54 ± 2.5	0.58 ^c^
BSI: hostility, mean ± SD	53.94 ± 11.7	52.7 ± 10.56	0.74 ^a^
BSI: paranoid ideation, mean ± SD	51.24 ± 8.91	56.1 ± 8.23	0.10 ^a^
BSI: anxiety, mean ± SD	54.53 ± 8.95	55.15 ± 12.42	0.86 ^a^
BSI: depression, median ± IQR	55 ± 17	55 ± 8.75	0.33 ^c^
BSI: somatization, mean ± SD	54.18 ± 9.93	57.5 ± 10.56	0.33 ^a^

FXM = F.X. Mayr diet, VLCD = very low-calorie diet, BMI = body-mass-index, CRP = C-reactive protein, IL6 = interleukin-6, GSI = global severity index, PSS = perceived stress scale, MtoF = male-to-female gender-trait ratio, HRV = heart rate variability, ^a^ unpaired *t*-test, ^b^ unpaired *t*-test after log10 transformation, ^c^ unpaired Wilcox test, ^d^ chi-square test. GSI, PSS, BODI and BSI variables are presented as points from the respective psychometric test, MtoF is a ratio.

**Table 2:** Summary of longitudinal PERMANOVA models.

Model	R^2^	p.adj
time	0.031	<0.05
time * CPI	0.047	n.s.
time * diet	0.079	<0.05
time * diet + CPI	0.09	<0.05
time * diet + CPI + age	0.104	<0.05
time * diet + CPI + age + BMI	0.12	<0.05

CPI = clinical psychological intervention, BMI = body-mass-index, p.adj = *p*-value after adjusting for multiple testing, n.s. = not significant. *p*-value adjustment was performed via Benjamini–Hochberg’s method.

## Data Availability

Dataset available via https://github.com/luisebe/diet-mental-health-microbiome.

## Supplementary Materials

The following supporting information can be downloaded at: https://www.mdpi.com/article/10.3390/nu16162584/s1, Table S1: Baseline differences alpha diversity. Table S2: Variables of longitudinal PERMANOVA models featured in Table 2. Table S3: Baseline differences with adjusted *p*-values. Figure S1: Delta beta-diversity between subgroups. Figure S2: Longitudinal changes in relative abundance, aggregated on species level. Figure S3: fully-annotated variable plot from the sPLS2 model depicted in Figure 3. Figure S4: Loadings of sPLS-DA of PSS-change (left) and diet group (sPLS-DA from Figure 4). Figure S5: Longitudinal taxonomic changes on genus level, stratified according to diet and CPI subgroups, logFC labels according to adjusted *p*-value < 0.2 (Benjamini-Hochberg); CPI … clinical psychological intervention, logFC … log-fold change. Figure S6: Flowchart of the study. Created with BioRender.com.
