# Supplementary material for: The Effects of Caloric Restriction and Clinical Psychological Intervention on the Interplay of Gut Microbial Composition and Stress in Women
Source: Nutrients. 2024 Aug 6;16(16):2584. doi: 10.3390/nu16162584 (PMC11357322; doi:10.3390/nu16162584)
Supplement: Supplementary file 1 [file nutrients-16-02584-s001.zip › nutrients-3110432-supplementary.pdf]

The effects of caloric restriction and clinical psychological intervention on the interplay of gut microbial composition and stress in women

Luise Bellach[1], Alexandra Kautzky-Willer[1], Kathrin Heneis[1], Michael Leutner[1], Alexander Kautzky[2]

[1] Department of Internal Medicine III, Division of Endocrinology and Metabolism, Medical University of Vienna, 1090 Vienna, Austria.
[2] Department of Psychiatry and Psychotherapy, Medical University of Vienna, 1090 Vienna, Austria.

Supplements:

variable	VLCD	FXM	test	p_value	p.adj
Firm_Bact_ratio, mean ± SD	0.69 ± 0.42	0.5 ± 0.24	unpaired t.test after log10 tf	0,139	0,327
observed_Genus, mean ± SD	45.56 ± 12.59	41.16 ± 16.19	unpaired t.test after log10 tf	0,218	0,327
shannon_Genus, mean ± SD	3.29 ± 0.25	3.22 ± 0.36	unpaired t.test	0,485	0,485

Table S 1: Baseline differences alpha diversity

variable	Df	SumOfSqs	R2	F	unadj.p	model	adj.p	significance
Timepoint	1	0,22	0,031	2,379	0	time	0	< 0.05
Timepoint	1	0,22	0,031	2,434	0	time * diet	0	< 0.05
host_diet	1	0,254	0,036	2,806	0	time * diet	0	< 0.05
Timepoint:host_diet	1	0,082	0,012	0,91	0,043	time * diet	0,048	< 0.05
Timepoint	1	0,22	0,031	2,353	0	time * CPI	0	< 0.05
Treatment	1	0,076	0,011	0,811	0	time * CPI	0	< 0.05
Timepoint:Treatment	1	0,035	0,005	0,378	0,697	time * CPI	0,697	n.s.
Timepoint	1	0,22	0,031	2,428	0	time * diet + CPI	0	< 0.05
host_diet	1	0,254	0,036	2,8	0	time * diet + CPI	0	< 0.05
Treatment	1	0,076	0,011	0,837	0	time * diet + CPI	0	< 0.05
Timepoint:host_diet	1	0,082	0,012	0,908	0,039	time * diet + CPI	0,048	< 0.05
Timepoint	1	0,22	0,031	2,431	0	time * diet + CPI + age	0	< 0.05
host_diet	1	0,254	0,036	2,803	0	time * diet + CPI + age	0	< 0.05
host_age	1	0,097	0,014	1,067	0	time * diet + CPI + age	0	< 0.05
Treatment	1	0,077	0,011	0,846	0	time * diet + CPI + age	0	< 0.05
Timepoint:host_diet	1	0,082	0,012	0,91	0,044	time * diet + CPI + age	0,048	< 0.05
Timepoint	1	0,22	0,031	2,438	0	time * diet + CPI + age + BMI	0	< 0.05
host_diet	1	0,254	0,036	2,811	0	time * diet + CPI + age + BMI	0	< 0.05
Treatment	1	0,076	0,011	0,84	0	time * diet + CPI + age + BMI	0	< 0.05
host_age	1	0,097	0,014	1,079	0	time * diet + CPI + age + BMI	0	< 0.05
host_body_mass_index	1	0,11	0,016	1,217	0,034	time * diet + CPI + age + BMI	0,045	< 0.05
Timepoint:host_diet	1	0,082	0,012	0,906	0,045	time * diet + CPI + age + BMI	0,048	< 0.05

Table S 2: Variables of longitudinal PERMANOVA models featured in Table 2

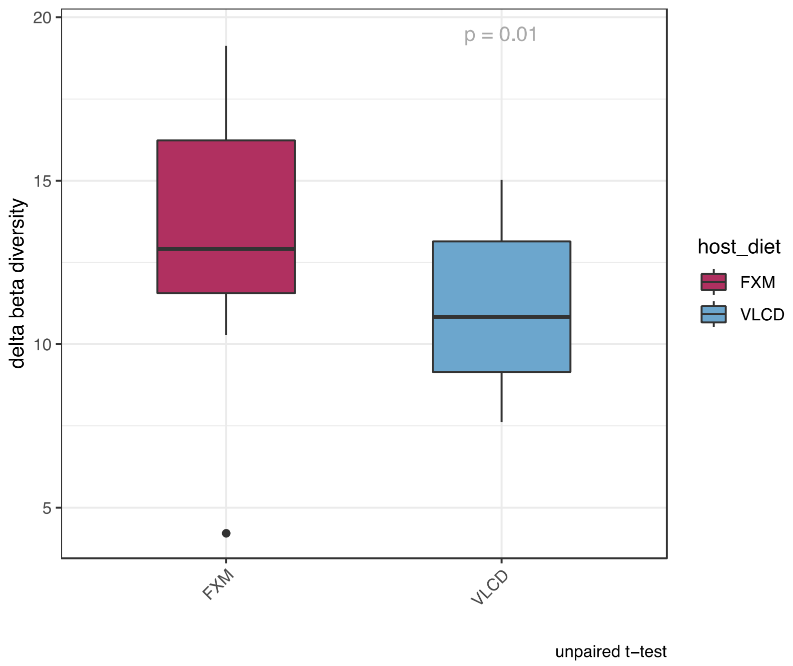

Figure S 1: Delta beta-diversity between subgroups

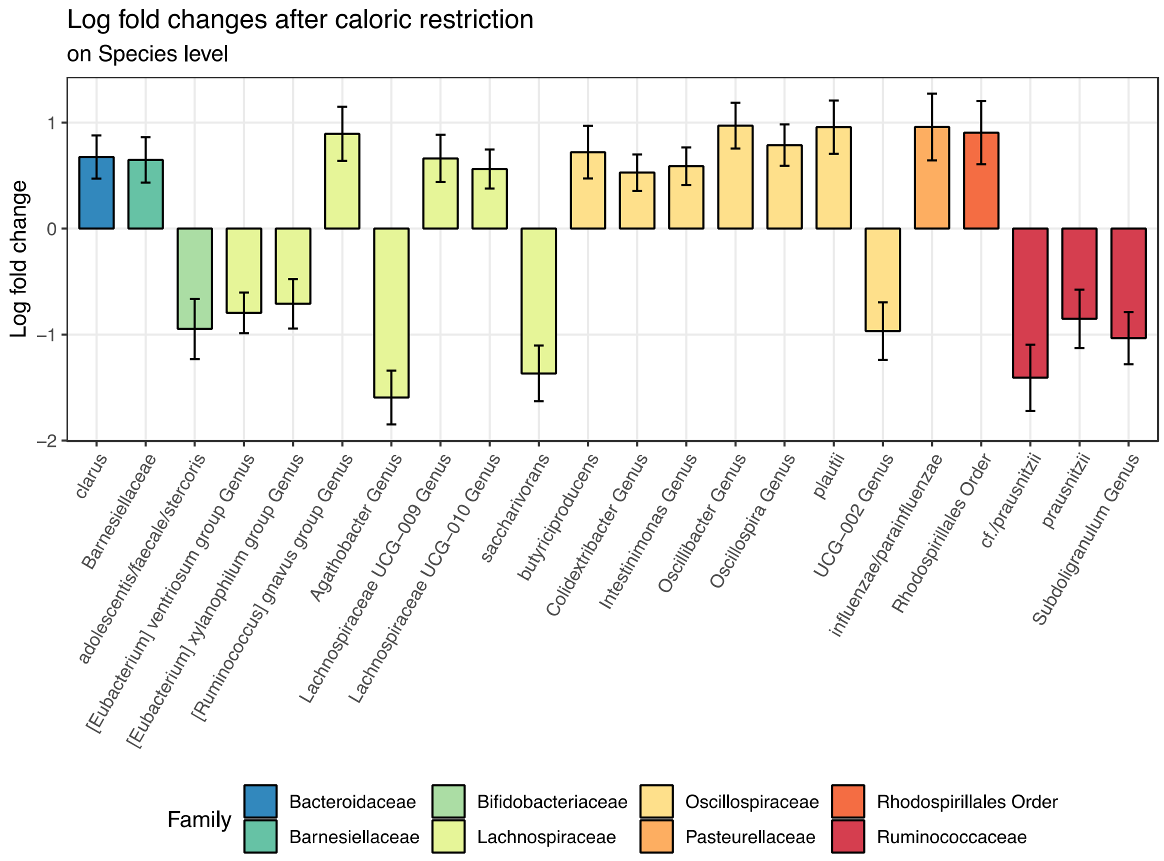

Figure S 2: Longitudinal changes in relative abundance, aggregated on species level

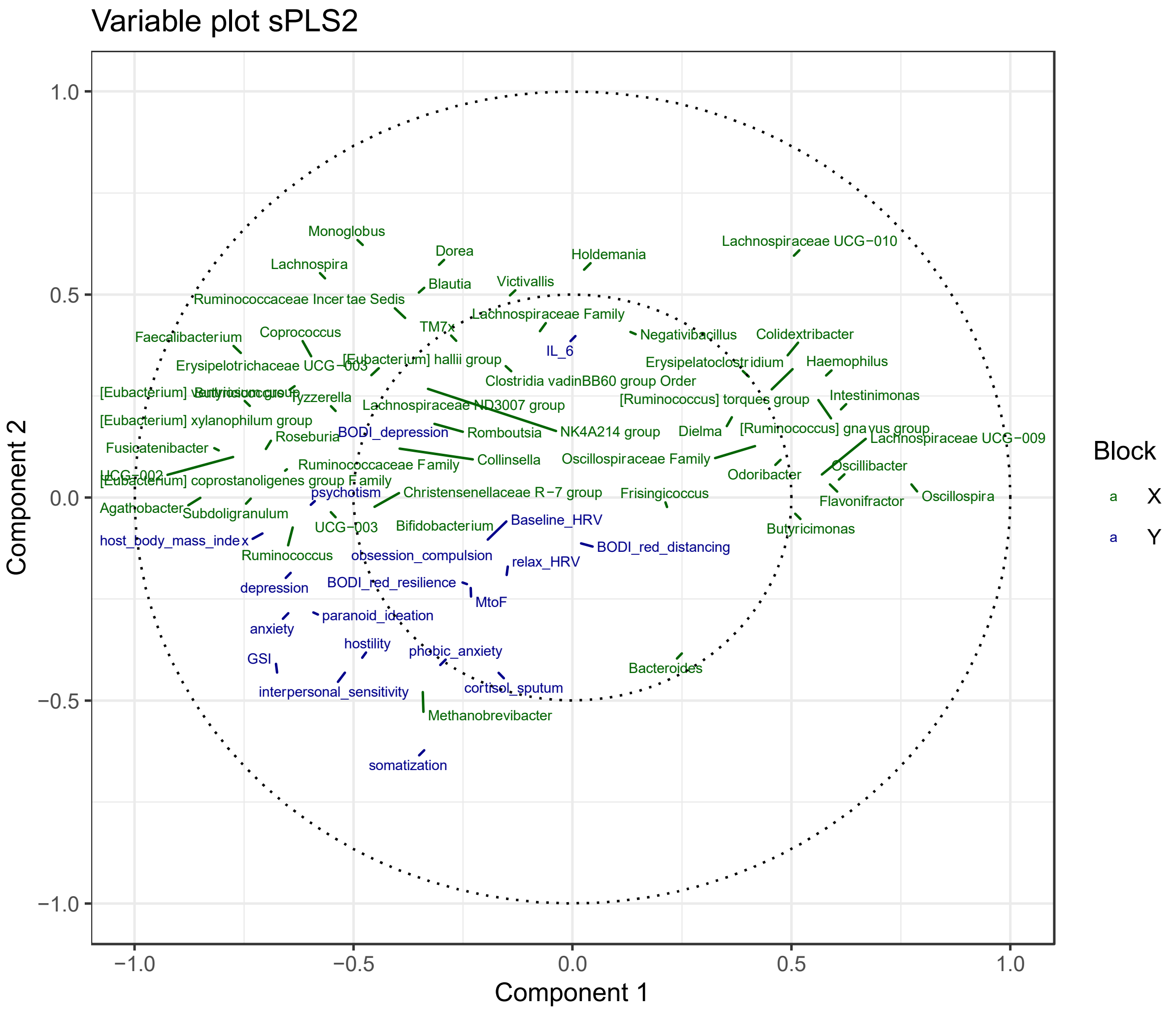

Figure S 3: fully-annotated variable plot from the sPLS2 model depicted in Figure 3

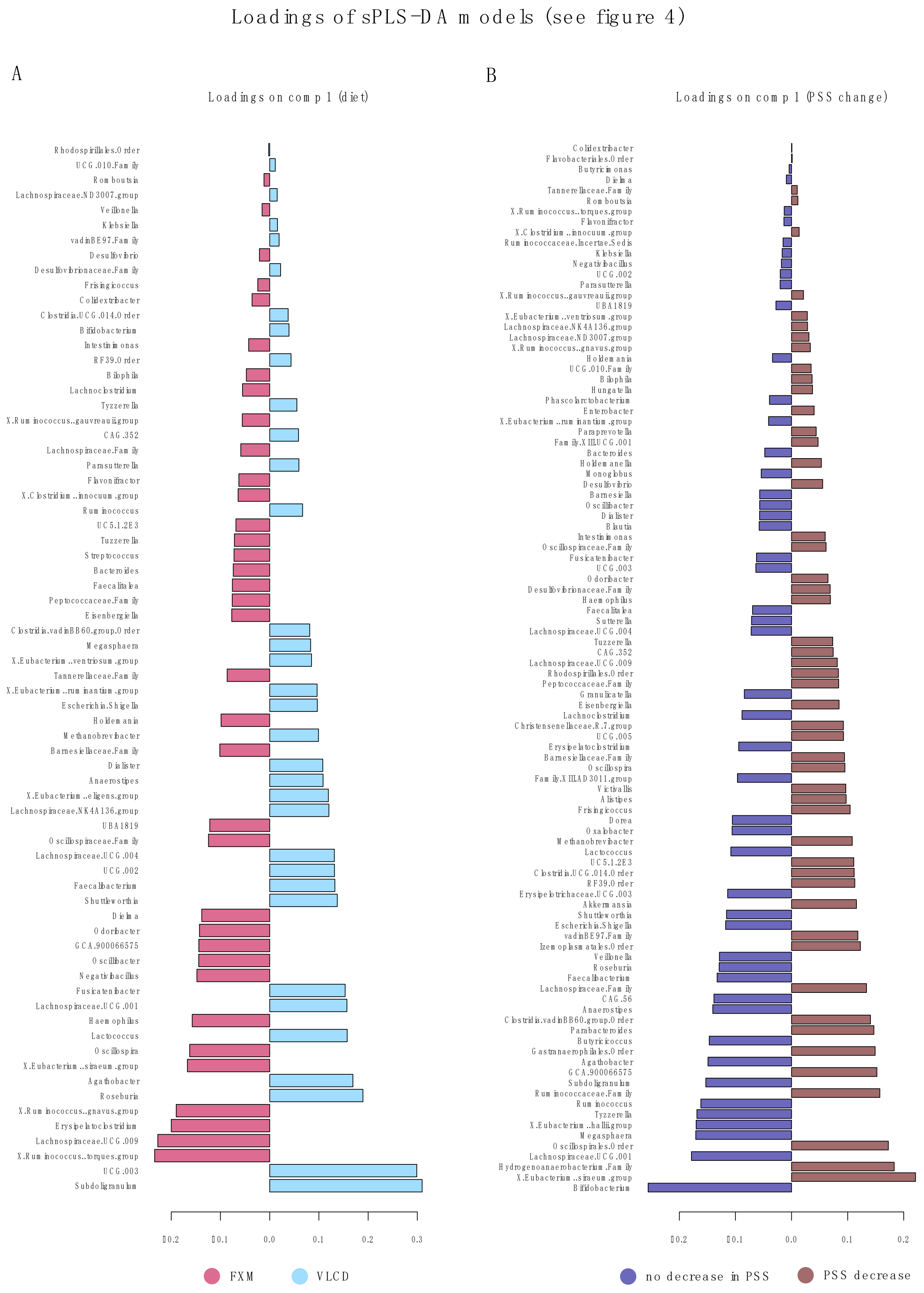

Figure S 4: Loadings of sPLS-DA of PSS-change (left) and diet group (sPLS-DA from Figure 4)

variable	VLCD	FXM	p-value	p.adj
age, mean ± SD	53.5 ± 10.82	53.55 ± 13.48	0.99 [a]	0.99
BMI, mean ± SD	28.69 ± 5.93	27.43 ± 5.93	0.47 [b]	0.87
CRP, mean ± SD	0.51 ± 0.49	0.28 ± 0.36	0.06 [b]	0.45
Cortisol in sputum, mean ± SD	0.46 ± 0.34	0.54 ± 0.32	0.23 [b]	0.93
IL6, median ± IQR	1.95 ± 3.32	1.87 ± 1.95	0.80 [c]	0.93
GSI, mean ± SD	54.06 ± 9.84	57.5 ± 10.72	0.32 [a]	0.95
PSS, mean ± SD	13.33 ± 3.73	16.94 ± 5.07	**0.04** [a]	0.48
MtoF, mean ± SD	1.11 ± 0.24	1.09 ± 0.24	0.86 [b]	0.93
BODI: dysfunctional compensation, median ± IQR	62 ± 96.5	45 ± 78	0.48 [c]	0.83
BODI: reduced resilience, mean ± SD	25.18 ± 15.3	35.95 ± 19.41	0.09 [a]	0.52
BODI: depression, median ± IQR	33.2 ± 22.9	26.9 ± 27.7	0.30 [c]	1.0
BODI: reduced distancing, median ± IQR	69.7 ± 35.73	53.8 ± 56.4	0.66 [c]	0.89
Baseline HRV, mean ± SD	12.21 ± 13.71	15.52 ± 13.59	0.36 [b]	0.78
Relaxed HRV, mean ± SD	16.07 ± 14.59	16.3 ± 9.74	0.62 [b]	0.87
Stressed HRV, mean ± SD	16.35 ± 16.12	17.03 ± 11	0.71 [b]	0.89
BSI: obsession-compulsion, mean ± SD	52.76 ± 9.09	55.85 ± 11.76	0.38 [a]	0.75
BSI: phobic anxiety, median ± IQR	45 ± 10	55 ± 16	**0.03** [c]	0.82
BSI: interpersonal sensitivity, mean ± SD	53.47 ± 9.64	55.35 ± 9.54	0.56 [a]	0.89
BSI: psychotism, median ± IQR	54 ± 10	54 ± 2.5	0.58 [c]	0.87
BSI: hostility, mean ± SD	53.94 ± 11.7	52.7 ± 10.56	0.74 [a]	0.89
BSI: paranoid ideation, mean ± SD	51.24 ± 8.91	56.1 ± 8.23	0.10 [a]	0.46
BSI: anxiety, mean ± SD	54.53 ± 8.95	55.15 ± 12.42	0.86 [a]	0.90
BSI: depression, median ± IQR	55 ± 17	55 ± 8.75	0.33 [c]	0.87
BSI: somatization, mean ± SD	54.18 ± 9.93	57.5 ± 10.56	0.33 [a]	0.79

Table S 3: Baseline differences with adjusted p-values

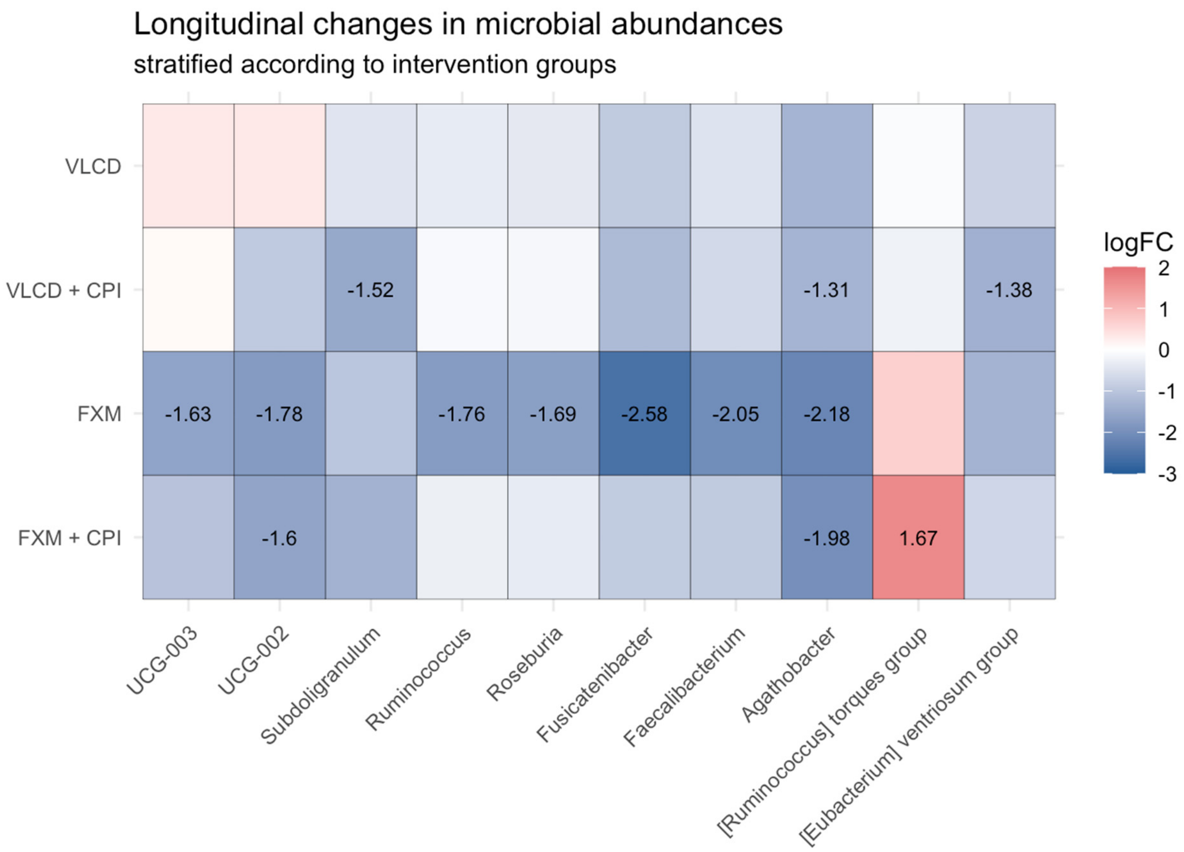

Figure S 5: Longitudinal taxonomic changes on genus level, stratified according to diet and CPI subgroups, logFC labels according to adjusted p-value < 0.2 (Benjamini-Hochberg); CPI … clinical psychological intervention, logFC … log-fold change

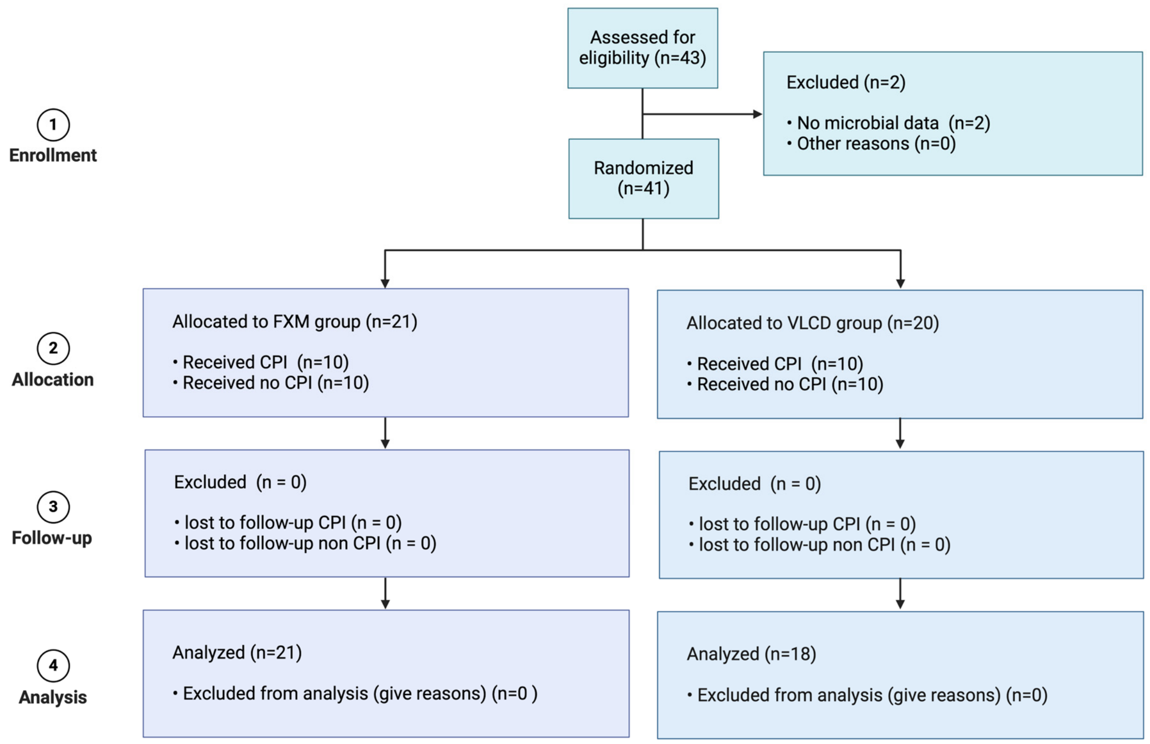

Figure S 6: Flowchart of the study. Created with BioRender.com
